# Cellular, Molecular, and Behavioural Sequelae of Early-Life Continuous Low-Dose-Rate Irradiation in Mice

**DOI:** 10.3390/cells15080711

**Published:** 2026-04-17

**Authors:** Feng Ru Tang, Hong Wang, Salihah Lau, Amanda Tan

**Affiliations:** Radiation Physiology Laboratory, Singapore Nuclear Research and Safety Institute, National University of Singapore, Singapore 118415, Singapore

**Keywords:** continuous low-dose rate, cumulative threshold dose, neurobehaviour, radiation, hippocampus, cellularity, miRNA/mRNA

## Abstract

The Fukushima nuclear accident highlighted that evacuation-related psychosocial harm can outweigh direct radiation risks, underscoring the need to define the health impacts of chronic low-dose-rate (LDR) radiation and evidence-based thresholds for intervention. This study investigated the effects of continuous, postnatal LDR gamma irradiation (1.2 mGy/h, cumulative dose: 5 Gy) in male mice. While no changes in body weight, hippocampal neurogenesis, or major glial and neuronal populations were observed, persistent DNA damage (γ-H2AX foci) in dentate gyrus granule cells occurred in both irradiated male and female mice. Irradiated male mice developed anxiety-like behaviour, a phenotype not observed in a previously published study of female mice subjected to an identical irradiation protocol. Molecular profiling revealed two novel, dysregulated miRNA/mRNA axes in the hippocampus linking DNA damage to behaviour: a maladaptive miR-466i-5p/Tfcp2l1 pathway associated with genomic instability, and a potentially adaptive miR-101a-5p/BMP6 pathway promoting neuronal survival. Venn analysis further identified miR-124b-3p and novel-miR489-3p as conserved exposure biomarkers, altered in both the hippocampus and blood of irradiated animals. Our results show that a high cumulative dose of chronic LDR induces markedly less severe hippocampal pathology than has been reported for equivalent acute doses. These findings support the concept of dose-rate-dependent threshold dose and contribute to the evidence base for developing countermeasures following nuclear incidents or other radiation exposures.

## 1. Introduction

The Fukushima nuclear accident underscored a critical lesson for radiation research: the severe indirect health consequences, including stress, anxiety, and over 2000 evacuation-related deaths, often outweighed the direct effects of radiation itself. This tragedy highlights the urgent need to understand the health impacts of chronic low-dose-rate (LDR) radiation, particularly its cumulative threshold doses [1,2]. Such knowledge is essential to inform evidence-based policies on evacuation timing and necessity.

Interestingly, some epidemiological and ecological studies suggest that chronic LDR exposure may not invariably be harmful. For instance, certain populations exposed to elevated natural background radiation or to low-dose-rate fallout after atomic bombings have shown lower cancer rates and increased longevity [3,4]. Furthermore, some wildlife species have thrived in radiologically contaminated environments [5]. Supporting this, studies on free-ranging meadow voles exposed to lifetime doses up to 10 Gy at 44 mGy/day revealed no clear impact on survival or reproduction [6]. Controlled laboratory investigations align with these observations. Chronic in utero gamma irradiation at dose rates below 20 mGy/day throughout gestation induced no obvious adverse health effects in B6C3F1 mice into adulthood and whole life [7,8]. Even at a higher dose rate of irradiation (100 mGy/day, cumulative dose: 1.8 Gy) during gestation, significant adverse effects in offspring were absent 1 year after radiation exposure, though subtle molecular changes in the hippocampus were detected [9]. Similarly, a previous study from our laboratory showed that postnatal female mice irradiated from day 3 (P3) for six months at 1.2 mGy/h (~5 Gy cumulative) exhibited no neurobehavioral or major hippocampal cellular alterations, aside from DNA damage in principal neurons and changes in hippocampal miRNA and mRNA [10]. This pattern differs markedly from acute irradiation with the same dose of 5 Gy but a high dose rate (3.3 Gy/min) on P3 mice, which produced substantial hippocampal pathology and miRNA alterations [11].

To further investigate the effects of chronic LDR exposure at high cumulative doses, and to extend our understanding to male subjects, this study examines postnatal male mice subjected to continuous LDR irradiation from P3. We present a detailed analysis of hippocampal cellular and molecular alterations and discuss these findings in the context of previously published results from female mice subjected to analogous experimental conditions.

## 2. Materials and Methods

### 2.1. Animal Model and Irradiation Protocol

BALB/c mouse pups and their dams were acquired from InVivos Pte. Ltd. (Singapore) on postnatal day 1 (P1) and maintained in the National University of Singapore’s Department of Comparative Medicine Facility. The animal experiment was performed according to the following timeline:


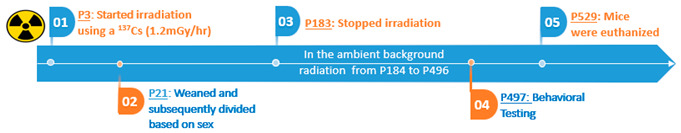


Starting on postnatal day 3 (P3), litters and dams underwent continuous whole-body γ-irradiation using a 137Cs source (G10-1-12 Gamma Beam Irradiator; Alpharetta, GA, USA) at a constant dose rate of ~1.2 mGy/h [12]. Exposure was maintained for 23.5 h daily, with a 30 min pause for animal care and cage servicing. Following weaning at P21, mice were separated by sex, and the chronic irradiation regimen was sustained at the same dose rate until postnatal day 183, yielding a total cumulative dose of approximately 5 Gy. Animals were then moved to a conventional housing facility with ambient background radiation levels.

Cumulative Dose Calculation: The total exposure period spanned 180 days (postnatal day 3 to 183). At a nominal dose rate of 1.2 mGy/h for 23.5 h/day, the calculated cumulative dose was: 1.2 mGy/h × 23.5 h/day × 180 days = 5076 mGy (5.08 Gy).

Dosimetry Monitoring: To monitor the radiation dose rate received by each cage, Nanodot dosimeters (LANDAUER, Glenwood, IL, USA) were placed inside 5 mL sterile tubes and positioned alongside the food pellet on the wire cage lid. This ensured that the dosimeters were exposed to the same radiation field as the animals. Cumulative doses were measured over multiple time intervals, and the dose rate was calculated as: dose rate = cumulative dose/(number of days × 23.5 h/day). Any variation from the target 1.2 mGy/h was corrected by adjusting cage positions within the rack to maintain uniformity. Dose Uniformity Assessment: The uniformity of the dose rate across the irradiator’s exposure field was assessed using a MAX 4000Plus Electrometer (Standard Imaging, Middleton, WI, USA), which confirmed a dose rate variation measured by Nanodots. This ensured that all animals received consistent exposure regardless of cage position. The study protocol received approval from the National University of Singapore’s Institutional Animal Care and Use Committee (IACUC Protocol #R20-0220).

A cohort of 13 mice was used. Male pups used in this study were derived from multiple litters: control mice (*n* = 7) from 4 dams (1 male from a litter of 7 pups, 1 male from a litter of 6 pups, 2 males from a litter of 7 pups, and 2 males from a litter of 3 pups. The remaining pups in these litters were female), and experimental mice (*n* = 6) from 3 dams (2 males from a litter of 7 pups, 3 males from a litter of 7 pups, and 1 male from a litter of 3 pups; the remaining pups in these litters were female). Due to the limited number of male pups per litter, all available males were included, and a split-litter design was not feasible. The small and unbalanced group sizes precluded statistical modelling of litter effects, which we acknowledge as a limitation of this study.

Animals were maintained under a 12 h light/dark cycle at 22 °C with ad libitum access to food and water. Cages were changed weekly, and health was monitored daily. Weekly weight measurements were collected for the first four weeks. This schedule was reduced to biweekly for the next eight weeks, with periodic assessments continuing until the time of sacrifice.

### 2.2. Behavioural Testing

At P497, mice (control: *n* = 7, irradiated: *n* = 6) underwent behavioural assessments. Group assignments were coded by the Principal Investigator, who was not involved in data collection. All experimenters were blinded to group allocation during behavioural scoring.

Animal behaviour was recorded and analysed by ANY-maze v 7.10 (ANY-maze, Wood Dale, IL, USA).

#### 2.2.1. Open Field Test (OFT)

Mice were allowed to explore a 50 × 50 cm opaque arena for 30 min. Time/distance in centre vs. periphery zones was tracked using ANY-maze.

#### 2.2.2. Elevated Plus Maze (EPM)

Mice were placed on a maze (50 cm height; 30 × 5 cm open/closed arms) for 10 min. Time/distance in open/closed arms was quantified.

#### 2.2.3. Tail Suspension Test (TST)

Mice were suspended by tape for 6 min. Immobility (≥2 s without limb movement) was recorded as a depression-like behaviour indicator.

#### 2.2.4. Forced Swim Test (FST)

Mice swam for 8 min in a 20 cm-diameter cylinder (24–26 °C water). Immobility time (≥2 s) was recorded.

### 2.3. Tissue Collection

At P529, mice were euthanised via CO_2_ asphyxiation. Blood was collected by cardiac puncture; 0.5 mL aliquots were stored in RNAlater (Thermo Fisher Scientific, Waltham, MA, USA) at −80 °C. Brains were dissected sagittally: left hippocampi were frozen (−80 °C) for RNA extraction; right hemispheres were fixed in 4% paraformaldehyde (24 h), then cryoprotected in 30% sucrose/0.1 M phosphate buffer (pH 7.4) for immunohistochemistry.

### 2.4. Immunohistochemistry (IHC)

Fixed hemispheres were sectioned sagittally (40 μm) using a Leica CM1950 cryostat (Leica Biosystems, Nussloch, Germany). Every sixth section was collected for IHC (6 series/antibody). Free-floating sections underwent peroxidase quenching (3% H_2_O_2_) and animal-free serum blocking. Sections were then incubated with rabbit primary antibodies overnight at 4 °C with the following concentrations: DCX (1:500, Santa Cruz, CA, USA), NeuN (1:1000, Invitrogen, Waltham, MA, USA), PDGFRα (1:200, Cell Signaling, MA, USA), GFAP (1:200, Cell Signaling, Danvers, MA, USA), IBA1 (1:200, Cell Signaling, MA, USA), γ-H2AX (1:200, Cell Signaling, MA, USA). After washing with phosphate-buffered saline with 0.1% Triton-X 100, sections were incubated in goat anti-rabbit secondary antibody, then ABC reagent (Vector Laboratories Inc., Burlingame, CA, USA), and reacted with in 3,3′-diaminobenzidine (DAB) peroxidase substrate (Vector Laboratories Inc., Burlingame, CA, USA). The sections were then washed, mounted, counterstained, and covered.

Quantification: Seven control and six experimental mouse samples were used for IHC examination; 7–9 sections/animal were imaged using a Leica DMi6 microscope. Stereologer 2000 software (Stereology Resource Centre, Tampa, FL, USA) was used to quantify NeuN, PDGFRα, and GFAP immunopositive cells in the hilus and IBA1 immunopositive cells in the hilus and stratum granulosum (cells/mm^3^). DCX immunopositive cells in the subgranular zone were normalised to length (cells/mm). Brightfield DAB-stained γ-H2AX foci were counted using a Leica DMi6 microscope with a 40× objective. Foci measuring approximately 2 µm in diameter were counted within the stratum granulosum and are presented as foci per mm^2^. We intentionally selected DAB immunohistochemistry over fluorescence for several practical reasons: (1) DAB-stained sections are permanently preserved and do not suffer from photobleaching, which is particularly advantageous given our large sample size; (2) this method eliminates the time constraints associated with fluorescence imaging, where signal decay can occur during extended counting sessions; and (3) DAB staining provides stable, high-contrast signals that facilitate consistent manual scoring across multiple experimental batches.

### 2.5. RNA Extraction from Hippocampus and Whole Blood

Total RNA was isolated from both hippocampus tissue and whole blood samples.

For hippocampus tissue (control: *n* = 3, irradiated: *n* = 3), samples were homogenized in QIAzol Lysis Reagent (Qiagen, Hilden, Germany). Following chloroform phase separation, RNA was purified from the aqueous phase using the miRNeasy Mini Kit (Qiagen, Hilden, Germany) according to the manufacturer’s instructions and eluted in RNase-free water.

For whole blood (control: *n* = 3, irradiated: *n* = 3), blood preserved in RNAlater was processed using the Mouse RiboPure^TM^-Blood RNA Isolation Kit (Thermo Fisher Scientific, Waltham, MA, USA). This procedure involved a phenol–chloroform extraction followed by purification through a vacuum filtration-based column system.

### 2.6. mRNA and miRNA Sequencing (miRSeq and mRNA-Seq)

RNAs from hippocampal and blood samples (*n* = 3 per group for each tissue type) were sent to BGI (Beijing, China) for complementary DNA (cDNA) library construction and subsequent sequencing on the DNBSEQ platform.

#### 2.6.1. mRNA-Seq Library Preparation and Sequencing

For mRNA sequencing, total RNA was denatured, and messenger RNA was enriched using oligo(dT) magnetic beads. The enriched mRNA was then fragmented, and first-strand cDNA was synthesized. Second-strand cDNA synthesis was performed with the incorporation of dUTP. The resulting double-stranded cDNA underwent end repair and 3′ adenylation before the ligation of sequencing adaptors. The libraries were then amplified by PCR. To prepare for sequencing, single-stranded circular DNA templates were generated, from which DNA nanoballs (DNBs) were produced via rolling circle amplification. These DNBs were loaded into patterned nanoarrays and sequenced using combinatorial Probe-Anchor Synthesis (cPAS).

#### 2.6.2. miRNA-Seq Library Preparation and Sequencing

For microRNA (miRNA) sequencing, RNA samples were sequentially ligated to 3′ and 5′ adapters. Following reverse transcription and PCR amplification, the products were size-fractionated and purified by PAGE gel electrophoresis and dissolved in EB buffer. Subsequently, single-stranded circular DNA templates were generated, amplified via rolling circle replication to form DNBs, loaded into patterned nanoarrays, and sequenced using cPAS.

In this project, we sequenced 3 samples from each of the blood and hippocampus of the control and experimental groups. Sequencing depth: Small RNA Sequencing: Blood Raw Tag Count (M) ranging from 26.04 to 27.62; Clean Tag Count (M) ranging from 25.28 to 26.76; Clean (%): 96.71 to 97.12; Genome Mapping (%): ranging from 96.71 to 97.84; and miRNA Annotation Rate (%): ~30.97. Hippocampal Raw Tag Count (M) ranging from 25.49 to 26.44; Clean Tag Count (M) ranging from 24.89 to 25.77; Clean (%): 97.12 to 97.72; Genome Mapping (%): ranging from 91.89 to 94.32; and miRNA Annotation Rate (%): ~30.97. mRNA Sequencing: Blood Raw Tag Count (M) ranging from 44.79 to 49.76; Clean Tag Count (M) ranging from 44.71 to 49.23; Clean (%): 91.33 to 93.93; Genome Mapping (%): ranging from 98.50 to 98.91; Gene Mapping (%): 85.38 to 87.91; and miRNA Annotation Rate (%): ~30.97. Hippocampal Raw Tag Count (M) ranging from 25.49 to 26.44; Clean Tag Count (M) ranging from 24.89 to 25.77; Clean (%): 97.12 to 97.72; Gene Mapping (%): 68.76 to 71.42; and mRNA Annotation Rate (%): ~30.97.

For the normalization approach, we used RSEM (Version: v1.2.8); Parameters: —p 8 —forward-prob 0.5 —paired-end, “URL (accessed on 8 August 2025, http://deweylab.biostat.wisc.edu/rsem/rsem-calculate-expression.html)” [12]. DESeq2 was used with default parameters, and no additional parameter adjustments were applied. The DESeq2 method is based on the negative binomial distribution described by Love et al. (2014) [13] to perform differential gene expression analysis. Parameters: Q value (Adjusted *p* value) ≤ 0.05.

Sequencing data analysed via DESeq2 (*p* < 0.05, FC > 1.5) indicated tissue-specific transcriptional responses to irradiation, with 375 mRNAs altered in the hippocampus and 252 in blood. miRNA profiling similarly identified 163 and 84 differentially expressed species in these tissues.

### 2.7. mRNA and miRNA Analyses Through Quantitative Real-Time PCR (qRT-PCR)

Gene and miRNA expression levels were validated using two-step quantitative reverse transcription PCR (qRT-PCR) on a QuantStudio 6 Real-Time PCR System. All primer sequences used for validation are provided in Supplementary Materials S1 and S2.

#### 2.7.1. mRNA Reverse Transcription and qPCR

First-strand cDNA for mRNA analysis was synthesized from RNA samples using the Maxima First-Strand cDNA Synthesis Kit (Thermo Fisher Scientific, Waltham, MA, USA). Each 20 µL reverse transcription reaction contained 1 µg RNA, 4 µL of 5× Reaction Mix, 2 µL Maxima Enzyme Mix, and nuclease-free water. The mixture was incubated at 25 °C for 10 min, 50 °C for 45 min, and 85 °C for 5 min.

Quantitative PCR was performed using the Maxima SYBR Green qPCR Master Mix. Amplification reactions (20 µL) consisted of 10 µL of 2× master mix, 2 µL of diluted cDNA, 2 µL of a pooled forward/reverse primer set (10× concentration), and 4 µL nuclease-free water. The thermal cycling protocol included an initial denaturation at 95 °C for 10 min, followed by 40 cycles of 95 °C for 15 s, 60 °C for 30 s, and 72 °C for 30 s. Relative target gene expression levels were calculated using the 2^–ΔΔCt^ method, with GAPDH serving as the internal control gene.

#### 2.7.2. miRNA Reverse Transcription and qPCR

Reverse transcription of mature miRNA was carried out using the miScript II RT Kit (Qiagen, Hilden, Germany). Each 20 µL reaction was assembled with 5 µL RNA, 2 µL reverse transcriptase mix, 4 µL of 5× HiSpec buffer, 2 µL of a 10× nucleic acid mix, and nuclease-free water. The incubation conditions were 37 °C for 60 min, followed by 95 °C for 5 min to inactivate the enzyme.

For real-time PCR detection of miRNAs, 20 µL reactions were prepared containing 10 µL of 2× miScript SYBR Green PCR master mix, 2 µL diluted cDNA, 2 µL of a 10× miScript universal primer, 2 µL of a specific primer for the target miRNA, and 4 µL nuclease-free water. The cycling parameters were: 95 °C for 15 min, followed by 40 cycles of 94 °C for 15 s, 55 °C for 30 s, and 70 °C for 30 s. Fluorescence data were collected during the extension step, and expression levels were standardized to the internal control small RNA miR-68.

### 2.8. Luciferase Reporter Assay Validation of miR-101a-5p, miR-466i-5p, and miR-466p-5p Targets

Potential target genes of three microRNAs (miR-101a-5p, miR-466i-5p, and miR-466p-5p), which exhibited significant expression changes following irradiation, were analysed and predicted using several online databases (TargetScan, miRanda, TarBase, miR2Disease, miRTarBase, miRecords, miRWalk). Based on this analysis, we selected specific targets for experimental validation: *Bmp6* (a target of miR-101a-5p); *Tfcp2l1 and Six3* (targets of miR-466i-5p); and *Igf2* (a target of miR-466p-5p) to confirm direct interaction.

The luciferase reporter assay was performed as previously described [14]. Briefly, fragments of the mouse 3′ untranslated regions (3′UTRs) *of Bmp6*, *Tfcp211*, *Six3*, and *Igf2* containing the respective binding sites for miR-101a-5p, miR-466i-5p, and miR-466p-5p were amplified by RT-PCR (primer sequences in Supplementary Materials S3). These amplified fragments were then directionally cloned into the XhoI and NotI restriction sites of the psiCHECK-2 plasmid, downstream of the Renilla luciferase gene. Firefly luciferase activity was used to normalise transfection efficiency.

To generate mutant controls, the seed regions for miR-101a-5p, miR-466i-5p, and miR-466p-5p within the *Bmp6*, *Tfcp211*, *Six3*, and *Igf2* 3′UTRs, respectively, were mutated using the Phusion Site-Directed Mutagenesis Kit (primer sequences in Supplementary Materials S3).

HEK293T cells were co-transfected with either the wild-type or mutant psiCHECK-2 constructs and the corresponding miRNA mimic (miR-101a-5p, miR-466i-5p, or miR-466p-5p) or a scrambled mimic control, using Lipofectamine 3000 according to the manufacturer’s protocol. Luciferase activity was quantified using the Dual-Luciferase^®^ Reporter Assay System 48 h after transfection.

### 2.9. Statistical Analysis

Body weight and behavioural data were analysed in the R statistical program (version 4.4.0). The body weight data were analysed using a two-way ANOVA, with treatment group as a between-subject factor and week measures as within-subject factors, using type-III sums of squares (car package).

Group comparisons (control vs. irradiated) for immunohistochemical and molecular (qRT-PCR) data were performed using the Student’s *t*-test. Data are presented as mean ± SEM. Statistical significance was assigned to results where *p* < 0.05. In the analysis of miRNA and mRNA sequencing via DESeq2, significant differential expression was identified using a dual cut-off of |log2FC| > 0.585 and an adjusted *p*-value < 0.05.

## 3. Results

### 3.1. Body Weight and Behavioural Changes

**Figure 1:**
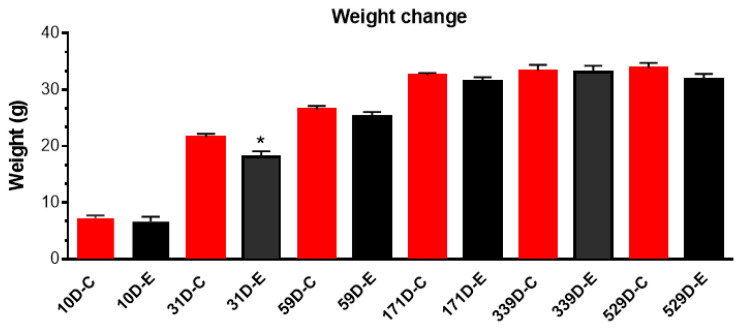
Body weight of mice following chronic γ-irradiation. Irradiated animals had a significantly lower body weight compared to controls across the experimental period, but not at individual time points of 10, 59, 171, 339, and 529 days of age (except 31 days) from the first day of irradiation at postnatal day 3, with both groups showing increasing body weight across time. The rate of weight gain did not differ between groups. Data are presented as mean ± SEM, * *p* < 0.05, control: *n* = 7; irradiated: *n* = 6.

Throughout the irradiation period, body weight changes in the irradiated group followed a specific pattern: it was stable at 10 days of age, showed a significant reduction at 31 days, but returned to a level not significantly different from the control group from 59 days onward. This non-significant difference persisted until the final measurement at 529 days of age (Figure 1). There is a statistically significant difference in body weight between the control and experimental groups when averaged across all time points. The animals’ weights changed significantly as they aged over the 529-day period. While the groups had different overall average weights, the pattern of weight gain over time (the growth trajectory) was statistically similar between the control and experimental groups. The effect of the experimental condition did not significantly alter how the animals gained weight across the days. For the body weight measured across the study period, there were significant main effects of treatment (F(1,52) = 8.19; *p* = 0.006), with irradiated animals having a lower body weight compared to controls, and day (F(4,52) = 90.40; *p* < 0.0001), with body weights increasing over time in both treatment groups (Figure 1). There was no significant treatment by day interaction (F(4,52) = 0.92; *p* = 0.459).

**Figure 2:**
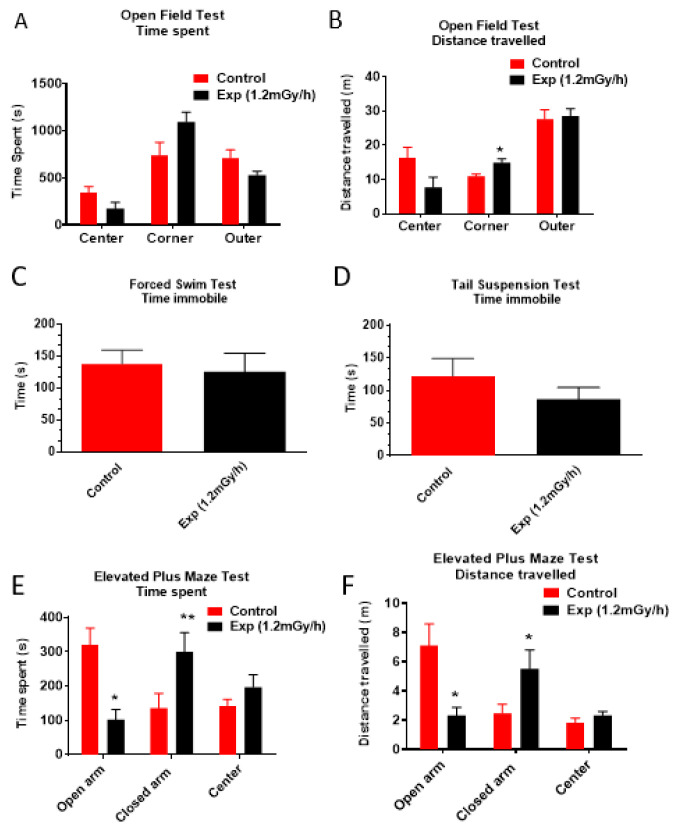
Neurobehavioural test. (**A**,**B**) Open Field Test: While no difference is observed in the time spent per zone ((**A**); *p* > 0.05), irradiated animals travelled a significantly greater distance in the corner regions (**B**), but this did not survive False Discovery Rate (FDR) correction (p_adj = 0.059), indicating no significant change. (**C**,**D**) Forced Swim Test (FST) (**C**) and tail suspension test (TST) (**F**) revealed no significant differences in immobility time between groups (*p* > 0.05). Elevated Plus Maze Test (**E**,**F**) irradiated animals spent significantly less time in the open arms ((**C**); * *p* < 0.01) and more time in the closed arms ((**E**); ** *p* < 0.05). Correspondingly, the distance travelled was significantly shorter in the open arms ((**F**), and longer in the closed arms (**F**). No differences were detected in the time or distance in the centre zone ((**E**,**F**); *p* > 0.05). Data are presented as mean ± SEM. Sample sizes: control: *n* = 7; irradiated: *n* = 6.

The Open Field Test (OFT) (Figure 2A,B), Forced Swim Test (FST) (Figure 2C), and Tail Suspension Test (TST) (Figure 2D) revealed no significant differences between groups. In the OFT, corner distance showed a nominal *p*-value of 0.010, but this did not survive False Discovery Rate (FDR) correction (p_adj = 0.059), indicating no significant change.

In the Elevated Plus Maze (EPM) (Figure 2E,F), the primary endpoint, and time spent in the open arms remained significant after FDR correction (p_adj = 0.019), with a very large effect size (Cohen’s d = 2.04) and a confidence interval that did not cross zero. This finding was supported by a secondary measure, open arm distance, which also survived FDR correction (p_adj = 0.032). In contrast, closed arm time and distance did not survive correction, indicating no significant changes in those measures.

Taken together, these results suggest that while irradiated mice exhibited increased anxiety-like behaviour, specifically in the EPM, no significant effects were observed for general exploratory activity or despair-like behaviour.

### 3.2. Immunohistochemical Assessment of Radiation-Induced Hippocampal Changes

**Figure 3:**
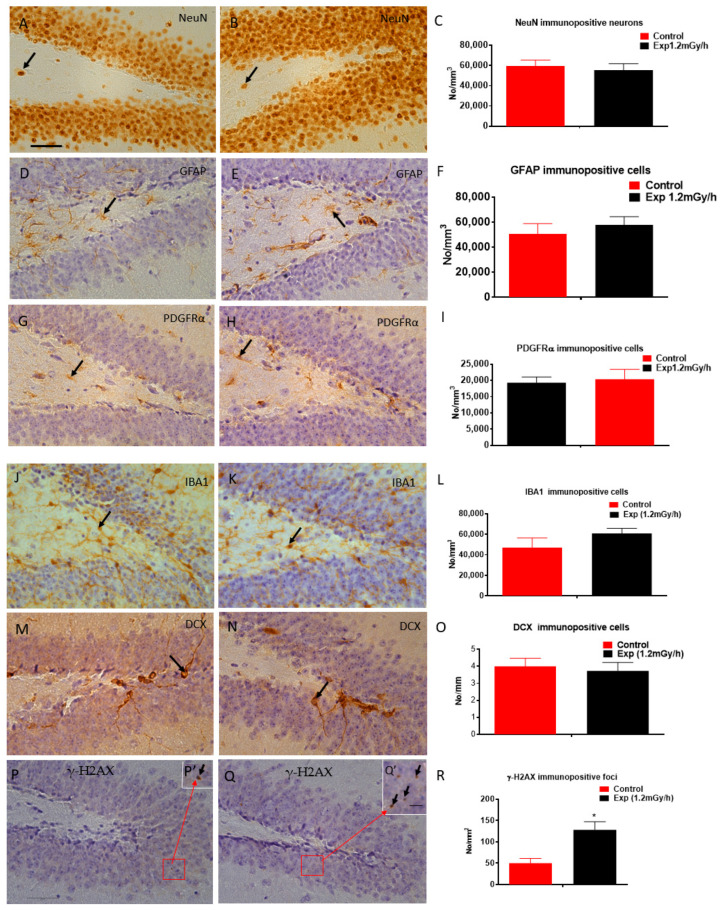
Immunohistochemical analysis. Representative micrographs of the hippocampal dentate gyrus show immunostaining for specific cell markers (**left panels**) with corresponding quantitative analyses (**right panels**). No significant differences were observed between control and irradiated groups in the number of (**A**–**C**) NeuN^+^ mature neurons (arrow in (**A**,**B**)), (**D**–**F**) GFAP^+^ astrocytes (arrow in (**D**,**E**)), (**G**–**I**) PDGFRα^+^ oligodendrocyte precursor cells (OPCs) in the hilus (arrow in (**G**,**H**)), (**J**–**L**) IBA1^+^ microglia in the hilus and granule cell layer (GCL) (arrow in (**J**,**K**)), or (**M**–**O**) DCX^+^ immature neurons in the subgranular zone (SGZ) (arrow in (**M**,**N**)). In contrast, (**P**–**R**) γ-H2AX immunostaining revealed a significant increase in DNA damage foci within the GCL following irradiation (* *p* < 0.01, (**R**)). Black arrows in higher-magnification insets (**P′**,**Q′**) within panels (**P**) and (**Q**) indicate representative foci. Scale bars: 100 µm in (**A**) applies to (**B**,**D**,**E**,**G**,**H**,**J**,**K**,**M**,**N**,**P**,**Q**); 5 µm in (**Q′**) applies to (**P′**). Data are presented as mean ± SEM. Sample sizes: control: *n* = 7; irradiated: *n* = 6.

The hippocampal cell landscape was largely preserved following irradiation. Quantitative immunohistochemistry showed no significant differences in the abundance of NeuN^+^ neurons, GFAP^+^ astrocytes, or PDGFRα^+^ precursor cells in the dentate gyrus hilus (Figure 3A–I). IBA1^+^ microglial distribution (Figure 3J–L) and DCX^+^ neuroblasts in the subgranular zone (Figure 3M–O) were also unaltered. Despite this cellular stability, a significant increase in γ-H2AX foci was evident in irradiated animals (*p* < 0.01; Figure 3P–R), revealing persistent genomic lesions at P529.

### 3.3. Differential Expression of mRNAs and miRNAs in the Hippocampus and Blood

**Figure 4:**
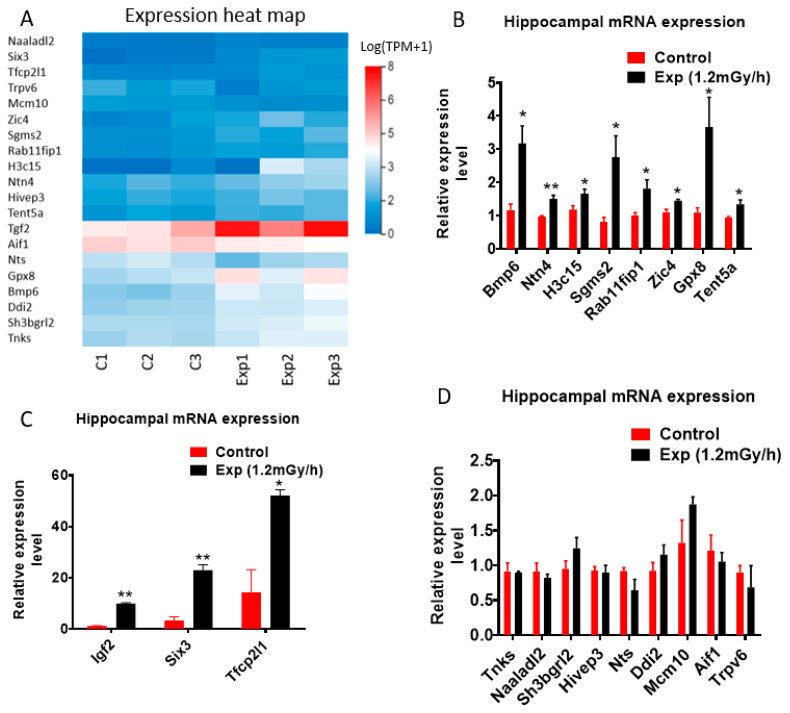
Gene expression from mRNA sequencing and qRT-PCR. (**A**) Heatmap of the expression of 20 genes selected. (**B**,**C**) qRT-PCR indicates significant upregulation of Bmp6, H3c15, Sgms2, Rab11fip1, Zic4, Gpx8, Tent5a, Tfcp2l1 (* *p* < 0.05), Ntn4, igf2, and Six3 (** *p* < 0.01). (**D**) qRT-PCR indicates no significant change of Tnks, Naaladl2, Sh3bgrl2, Hivep3, Nts, Ddi2, bMcm10, Aif1, and Trpv6 gene expression. Data are presented as mean ± SEM.

Targeted qRT-PCR validation focused on 20 hippocampal mRNAs related to genomic instability or neurological function (Figure 4A). This confirmed significant increases in *Bmp6*, *Ntn4*, *H3cl5*, *Sgms2*, *Rab11fip2*, *Zic4*, *Gpx8*, *Tent5a*, *Igf2*, *Six3*, and *Tfcp2l1* (Figure 4B,C), whereas a subset of nine genes showed no expression change (Figure 4D).

**Figure 5:**
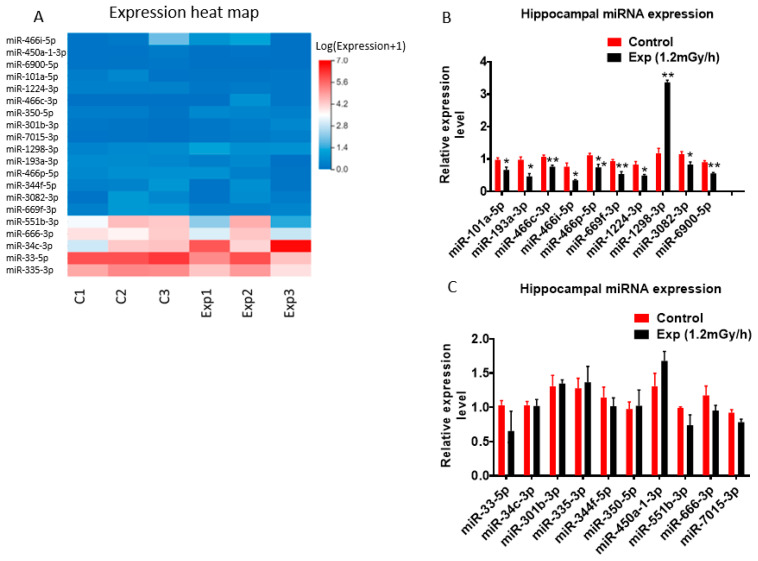
miRNA expression from miRNA sequencing and qRT-PCR. (**A**) Heatmap of the expression of 20 miRNAs selected. (**B**) qRT-PCR indicates significant upregulation of miR-1298-3p (** *p* < 0.01), and downregulation of miR-101a-5p, miR-193a-3p, miR-466i-5p, miR-1224-3p, miR-3082-3p (* *p* < 0.05), miR-466c-3p, miR-466p-5p, miR-669f-3p, and miR-6900-5p. (**C**) Irradiation does not induce a significant change in miR-33-5p, miR-34c-3p, miR-301b-3p, miR-335-3p, miR-344f-5p, miR-350-5p, miR-450a-1-3p, miR-666-3p, and miR-7015-3p. Data are presented as mean ± SEM.

For miRNAs, validation of a 20-target panel (Figure 5A) showed miR-1298-3p was upregulated, while nine others were downregulated (Figure 5B). The expression levels of nine additional miRNAs remained stable (Figure 5C).

**Figure 6:**
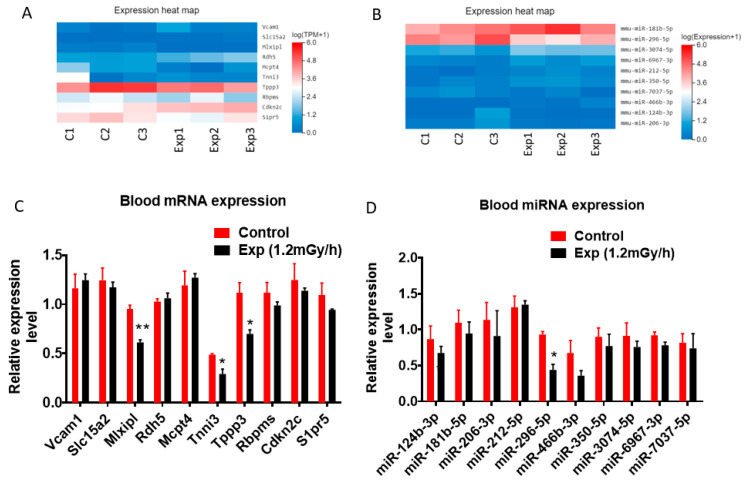
Blood mRNA and miRNA expression. (**A**,**B**) Heatmaps display the expression profiles of the 10 selected mRNAs and 10 miRNAs. (**C**) qRT-PCR validation confirms the significant downregulation of *Mlxipl* (** *p* < 0.01), *Tnni3* (* *p* < 0.05), and *Tppp3*. (**D**) qRT-PCR validation confirms the significant downregulation of miR-296-5p. Data are presented as mean ± SEM.

From 252 differentially expressed mRNAs and 84 miRNAs identified in blood samples, the top 10 most promising candidates from each group were selected for further analysis based on their expression levels and significance. Heatmaps display these selected mRNAs and miRNAs (Figure 6A,B). Subsequent qRT-PCR validation confirmed that, among the 10 mRNAs, 3 (*Mlxipl*, *Tnni3*, and *Tppp3*) were significantly downregulated (Figure 6C). Similarly, among the 10 miRNAs, only miR-296-5p showed significant downregulation (Figure 6D).

### 3.4. Experimental Validation of Direct miRNA-Target Gene Interactions

**Figure 7:**
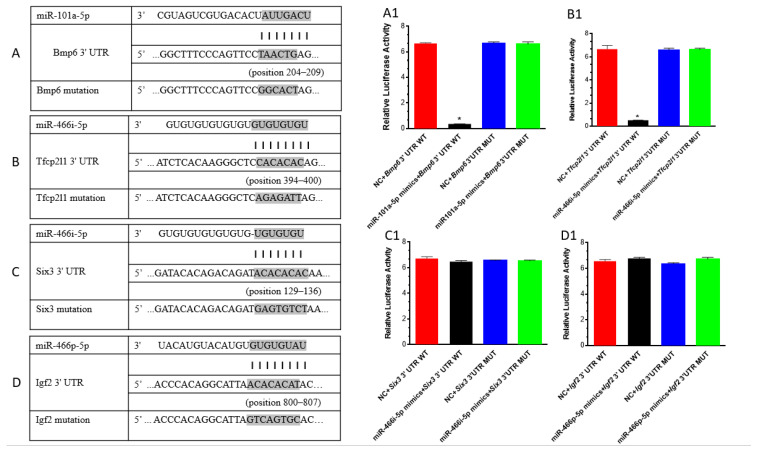
Interaction of miRNAs with the predicted targets examined by luciferase reporter assay. (**A**–**D**) Sequence alignment of putative miR-101a-5p binding sites in *Bmp6* 3′ UTRs (**A**), miR-466i-5p binding sites in *Tfcp2l1* 3′ UTR (**B**), miR-466i-5p binding sites in *Six3* 3′ UTR (**C**), miR-466p-5p binding sites in *Igf2* 3′ UTR (**D**), and the mutation sequences (highlighted in grey, each vertical line represents the specific paired binding between a microRNA (miR) and its corresponding 3′ untranslated region (3′UTR)), respectively. Activity of the luciferase gene linked to the 3′ UTR of Bmp6 (**A1**), *Tfcp2l1* (**B1**), *Six3* (**C1**), and *Igf2* (**D1**) mRNA. HEK293T cells were co-transfected with psiCHECK-2 constructed with 3′ UTR binding sites of miR-101a-5p (**A1**), miR-466i-5p (**B1**,**C1**), and miR-466p-5p (**D1**) mimic or scrambled mimic control. Luciferase and Renilla signals were measured 48 h after transfection. Data are expressed as mean ± SEM. * *p* < 0.001.

Potential targets of miR-101a-5, miR-466i-5p, and miR-466p-5p were identified according to 2.9 using multiple online databases. To validate predicted interactions of miR-101a-5 with Bmp6, miR-466i-5p with *Tfcp2l1 or Six3*, and miR-466p-5p with *Igf2*, the direct binding of miR-101a-5p to *Bmp6* and of miR-466i-5p to *Tfcp2l1* or Six3, miR-466p to *Igf2* was assessed using luciferase reporter assays. Putative binding sites within the 3′ UTR of each target were cloned into the psiCHECK-2 vector, including the following: mouse miR-101a-5p at positions 204–209 of *Bmp6* (Figure 7A), miR-466i-5p at positions 394–400 of *Tfcp2l1* (Figure 7B), positions 129–136 of *Six3* (Figure 7C), and miR-446p-5p at positions 800–807 of *Igf2* (Figure 7D).

Co-transfection of miR-101a-5p mimic with the *Bmp6* 3′ UTR reporter construct into HEK 293 cells resulted in a significant reduction in luciferase activity compared to the negative control (miR-NC). For the wild-type *Bmp6* 3′UTR construct (*Bmp6*-WT), miR-101a mimics significantly reduced luciferase activity compared to the mimics NC control (0.29 ± 0.03 vs. 1.00 ± 0.02, ~71% repression, *p* < 0.001) (Figure 7(A1)). The effect size was extremely large (Cohen’s d = 73.3), confirming a robust and specific interaction. This repressive effect was completely abolished in the seed-region mutant construct (*Bmp6*-Mut), where luciferase activity was comparable between miR-101a and control groups (0.99 ± 0.05 vs. 1.00 ± 0.03, *p* = 0.78), with a small effect size (Cohen’s d = 0.26) (Figure 7(A1)). These results demonstrate that *Bmp6* is a direct functional target of miR-101a via the predicted seed sequence. Similarly, for the wild-type *Tfcp2l1* 3′UTR construct (*Tfcp2l1*-WT), miR-466i-5p mimics dramatically reduced luciferase activity compared to the mimics NC control (0.08 ± 0.01 vs. 1.00 ± 0.09, ~92% repression, *p* < 0.001) (Figure 7(B1)). The effect size was extremely large (Cohen’s d = 14.0), confirming a robust and specific interaction. This repressive effect was completely abolished in the seed-region mutant construct (*Tfcp2l1*-Mut), where luciferase activity was comparable between miR-466i and control groups (1.01 ± 0.04 vs. 1.00 ± 0.04, *p* = 0.67), with a negligible effect size (Cohen’s d = −0.16). These results unequivocally demonstrate that *Tfcp2l1* is a direct functional target of miR-466i via the predicted seed sequence. For the wild-type *Six3* 3′UTR construct (*Six3*-WT), miR-466i-5p mimics produced a modest but statistically significant reduction in luciferase activity compared to the mimics NC control (0.96 ± 0.02 vs. 1.00 ± 0.04, ~4% repression, *p* = 0.042) (Figure 7(C1)). The effect size was large (Cohen’s d = 1.04), suggesting a consistent albeit small effect. However, for the seed-region mutant construct (*Six3*-Mut), miR-466i-5p mimics showed no significant difference from the control (1.00 ± 0.02 vs. 1.00 ± 0.01, *p* = 0.83), with a small effect size (Cohen’s d = 0.21). While the wild-type construct showed statistically significant repression, the biological relevance of a 4% reduction is uncertain, and *Six3* may not be a physiologically significant target of miR-466i-5p. For the wild-type *Igf2* 3′UTR construct (*Igf2*-WT), miR-466p mimics did not repress luciferase activity; rather, a modest but statistically significant increase was observed compared to the mimics NC control (1.03 ± 0.02 vs. 1.00 ± 0.04, +3%, *p* = 0.048). The effect size was large in the opposite direction (Cohen’s d = −1.00), indicating that *Igf2* is not a functional target of miR-466p under these conditions. For the seed-region mutant construct (*Igf2*-Mut), miR-466p-5p similarly produced a significant increase in luciferase activity compared to the control (1.06 ± 0.04 vs. 1.00 ± 0.03, +6%, *p* = 0.008) (Figure 7(D1)), with a very large effect size in the opposite direction (Cohen’s d = −1.52). These results indicate that despite bioinformatic predictions of binding, *Igf2* does not function as a direct target of miR-466p-5p, and the slight increase observed may reflect indirect effects or experimental variability. (For the exact sequences of inserted UTR fragments and the mutated seed-region changes, please refer to Supplementary Materials S4. For the luciferase assay results, refer to Supplementary Materials S5). Together, these results indicate that miR-101a-5p and miR-466i-5p directly target the 3′ UTR of *Bmp6* and *Tfcp2l1*, respectively, while *Six3* and *Igf2* are not functional targets of miR-466i-5p and miR-446p-5p, respectively, under these experimental conditions.

### 3.5. Venn Analysis of Differentially Expressed mRNAs and miRNAs in the Hippocampus and Blood of Male and Female Mice

**Figure 8:**
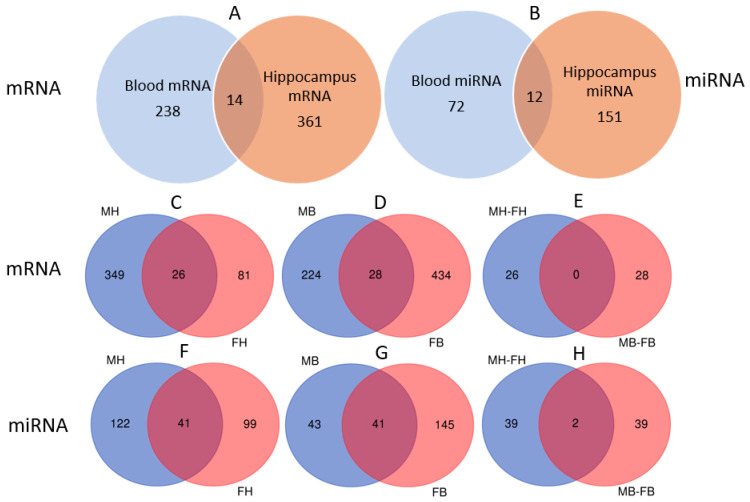
Venn analysis of differentially expressed mRNA and miRNA: (**A**,**B**) Male mice: 14 mRNAs (**A**) and 12 miRNAs (**B**) are differentially expressed in both the hippocampus and blood. (**C**–**E**) mRNAs across sexes and tissues: 26 mRNAs are altered in the hippocampus (**C**) and 28 in the blood (**D**) of both male and female mice. No mRNA is altered across all four groups, i.e., in both the hippocampus and blood of both sexes (**E**). (**F**–**H**) miRNAs across sexes and tissues: 41 miRNAs are altered in the hippocampus (**F**) and 41 in the blood (**G**) of both male and female mice. Two miRNAs, miR-124b-3p and novel-miR489-3p, are altered in both tissues of both sexes (**H**). MH: male hippocampus; FH: Female hippocampus; MB: male blood; FB: female blood.

In male mice, Venn analysis revealed 14 (Supplementary Materials S6) significantly altered mRNAs (Figure 8A) and 12 (Supplementary Materials S7) altered miRNAs (Figure 8B) common to both the hippocampus and blood.

A comparison across sexes showed 26 (Supplementary Materials S8) and 28 (Supplementary Materials S9) significantly altered mRNAs in the hippocampus (Figure 8C) and blood (Figure 8D) of both males and females, respectively. Notably, no single mRNA alteration was shared across all four groups—that is, in both the hippocampus and blood of both sexes (Figure 8E). Analysis of miRNAs across sexes identified 41 (Supplementary Materials S10) altered miRNAs in the hippocampus (Figure 8F) and 41 (Supplementary Materials S11) in the blood (Figure 8G) common to both males and females. Strikingly, two miRNAs, miR-124b-3p and novel-miR489-3p, were altered in both tissues and in both sexes (Figure 8H).

## 4. Discussion

The evacuation but not radiation-related deaths of more than 2000 residents after the Fukushima nuclear accident underscores the importance of research on the health effect of low-dose-rate radiation and related threshold dose, which are essential for making scientifically evidence-based policies on evacuation timing and necessity as wild and laboratories animal studies have consistently indicated that chronic LDR irradiation with cumulative high doses may not induce adverse health effects. In this study of the male Balb/c mice after a chronic LDR from postnatal day 3 to day 180 with a cumulative dose of 5Gy, we did not observe animal weight change. The dentate gyrus neurogenesis and other cellular changes, including mature neurons, oligodendrocyte precursor cells, astrocytes, and microglia, were not observed, but DNA damage occurred in the granule cells, which is consistent with our study on female mice. The irradiated male mice had no depression but developed anxiety, which was not observed in the female group.

### 4.1. Biological Effects of Chronic Low-Dose-Rate Irradiation: Behavioural Changes and Persistent DNA Damage

Our findings indicate that chronic LDR irradiation over six months increased anxiety-like behaviour in irradiated mice, as measured by the Open Field and Elevated Plus Maze tests, with no concomitant signs of depression-like behaviour in the Tail Suspension or Forced Swim tests. At the cellular level, immunohistochemical analysis demonstrated a persistent increase in γ-H2AX foci within the granule cells of the dentate gyrus, a marker of DNA damage. This suggests that chronic LDR exposure induces lasting genomic instability, consistent with reports following acute high-dose-rate exposure to an equivalent total dose [13].

In contrast to acute exposure paradigms [14], we observed no significant changes in hippocampal neurogenesis within the subgranular zone, hypoplasia of the stratum granulosum, microglial reactivity in the hilus or stratum granulosum, or alterations in the populations of mature neurons and oligodendrocyte precursor cells. This distinct outcome, where DNA damage occurs without triggering broader cellular pathology, aligns with observations from other research groups investigating chronic low-dose-rate exposure. For example, studies by Tanaka and colleagues [8,9] demonstrated that continuous in utero gamma irradiation at low dose rates produced minimal adverse effects despite cumulative doses that would be highly damaging if delivered acutely. Similarly, work by Mihok [7] on wild meadow voles showed that low-dose-rate gamma radiation at approximately 44 mGy/day, accumulating mean doses in the range of 4–6 Gy over a lifetime, had no clear impacts on survival or reproduction.

The contrast between our findings and those from acute irradiation studies [13,14] underscores a critical principle: the adverse biological effects of chronic LDR exposure at high cumulative doses are markedly less severe than those of acute exposure at the same total dose. This pattern has been corroborated by multiple laboratories, including research on Chernobyl wildlife [15,16,17] and those animals exposed to low-dose-rate radiation [18]. These converging lines of evidence from both field and laboratory studies suggest that there may exist cumulative threshold doses for different chronic low-dose-rate radiation exposures, or threshold dose rates for different cumulative doses.

The primary observed effect in our model was persistent DNA damage in a specific neuronal population, without the cascading cellular dysfunction typically seen after acute irradiation. This finding aligns with studies from other groups examining radiation-induced DNA damage responses. For instance, work by Löbrich and colleagues has demonstrated that DNA repair kinetics and cellular outcomes differ substantially between acute and protracted exposures, with low dose rates allowing more efficient repair and reduced fixation of damage [19].

### 4.2. Divergent Molecular Pathways in Radiation-Induced Hippocampal Injury

Our study delineates two novel and distinct molecular axes, miR-466i-5p/Tfcp2l1 and miR-101a-5p/BMP6, which are dysregulated in the hippocampus following chronic low-dose-rate radiation. Both pathways connect the persistent DNA damage observed in dentate gyrus granule cells to the specific neurobehavioral phenotype of increased anxiety, yet they appear to mediate opposing cellular programs: one maladaptive and the other potentially protective.

#### 4.2.1. The miR-466i-5p/Tfcp2l1 Axis: A Maladaptive Shift Leading to Genomic Instability

Radiation-induced suppression of miR-466i-5p leads to the derepression of its direct target, Tfcp2l1, a transcription factor involved in stemness and DNA repair. This dysregulation occurs in post-mitotic neurons, where its normal functions may become detrimental. The downregulation of miR-466i-5p, a known pro-apoptotic miRNA, may disrupt the normal clearance of damaged cells, contributing to apoptosis in various tissue insult models [20,21,22,23,24,25]. KCNQ1 overlapping transcript 1 (Kcnq1ot1) sponges miR-466i-5p to upregulate TEA domain transcription factor 1, leading to cardiomyocyte injury during acute myocardial infarction [20]. The positive correlation of miR-466i-5p with Snai2, Cdc27, and Ngfr contributes to chronic intermittent hypoxia-exaggerated post-myocardial infarction remodelling [21]. In myocardial ischemia/reperfusion (I/R) injury, LincRNA-p21 positively regulates the expression of nuclear receptor subfamily 4 group A member 2 by sponging miR-466i-5p, which promotes cardiomyocyte apoptosis, suggesting a novel LincRNA-p21/miR-466i-5p/Nr4a2 pathway for myocardial I/R injury [22]. Additionally, lncMN2-203 facilitates motor neuron differentiation by sponging miR-466i-5p and upregulating its targets involved in neuronal differentiation and function [23]. In a heatstroke mouse model and a heat-stressed neuronal cellular model using HT22 cells, Zhu et al. (2022) demonstrated that upregulated microglial exosomal miR-466i-5p is taken up by neurons, inducing neuronal apoptosis by targeting Bcl-2 and activating caspase-3 [24]. This implicates the Bcl-2/caspase-3 pathway in miR-466i-5p-related apoptosis. Bioinformatics analysis and dual-luciferase reporter assays reveal that long noncoding RNAs bind to miR-466i-5p, with caspase 8 identified as a target of this miRNA. The lncRNA-PEAK1/miR-466i-5p/caspase 8 axis is closely related to neuronal apoptosis following intracerebral haemorrhage [25].

Concurrently, aberrant Tfcp2l1 upregulation may initiate an ineffective or inappropriate “stem-cell-like” repair program [26]. We propose that this axis represents a maladaptive response, leading to failed repair mechanisms and the persistence of genomically unstable granule cells. This instability compromises the integrity of hippocampal circuits and drives anxiety-like behaviour.

#### 4.2.2. The miR-101a-5p/BMP6 Axis: An Adaptive, Contained Survival Response

In contrast to other pathways, the radiation-induced downregulation of miR-101a-5p and the subsequent upregulation of its target, BMP6, appear to represent a targeted, adaptive response. BMP6 is a hippocampus-enriched factor known for its roles in neuroprotection and synapse formation [27,28,29,30,31]. It is highly expressed and predominantly neuron-specific within the hippocampus, where it localises to and near DCX-positive neurogenic cells in the SGZ of the dentate gyrus. BMP6 is crucial for neuronal maturation and synaptic development [27,30].

Beyond its developmental roles, BMP6 exhibits significant neuroprotective properties across various injury models. For example, mild ischemia triggers the release of BMP6 from neurons into the interstitial space, indicating its involvement in enhancing adult neuronal resilience [28]. Additionally, BMP6 potentiates the survival effects of neurotrophins on cholinergic neurons during metabolic stress [29]. Following traumatic brain injury, BMP6 expression is notably upregulated in hippocampal and cortical neurons by day 3, and becomes prominent in reactive astroglia surrounding the lesion site by 48 h. This suggests a dual role for BMP6 in both neuronal protection and the astroglial response [32]. However, this protective role may become maladaptive under chronic conditions. Elevated levels of BMP6 are observed in the hippocampi of Alzheimer’s Disease (AD) patients and APP transgenic mice, where increased BMP6 correlates with impaired hippocampal neurogenesis. This dysregulation implicates BMP6 in AD-related neurogenic deficits and highlights its potential as a therapeutic target [30,31].

We hypothesise that the miR-101a-5p/BMP6 axis functions as a damage-containment mechanism. By promoting granule cell resilience and favouring repair over apoptosis, enhanced BMP6 signalling may sustain the viability and synaptic function of neurons harbouring low-level DNA damage. This model accounts for the coexistence of persistent DNA lesions without overt cell loss, neurogenic disruption, or glial activation, thereby representing an attempt to preserve circuit homeostasis.

In conclusion, chronic radiation exposure concurrently triggers at least two regulatory pathways in hippocampal granule cells. The miR-466i-5p/Tfcp2l1 axis may propagate injury by allowing damaged cells to accumulate, while the miR-101a-5p/BMP6 axis may promote survival and functional maintenance. This dual-response model clarifies the subtle pathogenesis of radiation-induced neurotoxicity, where a balance between such opposing signals could determine long-term outcomes. Both axes present promising but distinct therapeutic targets: suppressing the maladaptive Tfcp2l1 signal versus augmenting the protective BMP6 pathway. Future studies that manipulate these interactions in vivo are essential to validate their causal roles and to explore their potential to mitigate the neurocognitive sequelae of radiation exposure.

### 4.3. miR-124b-3p and miR-489-3p as Conserved Biomarkers of Chronic Radiation Exposure in the Hippocampus and Blood

mRNA [33,34] and miRNA [35,36,37] have been considered as promising biomarkers for low-dose/dose-rate radiation exposure. Our analysis reveals distinct patterns of mRNA and miRNA expression in the hippocampus and blood of mice following chronic LDR radiation. In male mice, we identified 14 mRNAs and 12 miRNAs that were significantly altered in both tissues. This co-occurrence suggests that peripheral alterations in blood-borne mRNAs and miRNAs may serve as accessible biomarkers for monitoring corresponding molecular changes in the hippocampus.

When compared with our study on female mice [10], we observed a significant number of altered transcripts common to both sexes within each tissue: 26 mRNAs and 41 miRNAs in the hippocampus, and 28 mRNAs and 41 miRNAs in the blood. This indicates that a substantial portion of the molecular response to chronic low-dose-rate radiation is conserved across genders.

Notably, while no single mRNA was altered across all four conditions (both tissues in both sexes), two miRNAs met this criterion: miR-124b-3p and novel-miR489-3p. The consistent dysregulation of these two miRNAs in both the hippocampus and blood of irradiated male and female mice positions them as particularly promising candidate biomarkers. They may enable the detection of chronic radiation exposure at a low-dose rate (1.2 mGy/h) and a cumulative dose of 5 Gy, irrespective of biological sex or tissue source. Interestingly, when 8-week-old, ApoE-deficient (ApoE^−/−^) female mice were chronically whole-body irradiated (20 mGy/d, cumulative dose of 3 Gy) for 150 days, it was indicated that the absence of ApoE^-/-^ influenced synaptic functionality and integration by interfering with the regulation of miR-34a, miR-29b, and miR-128b, leading to the downregulation of synaptic markers PSD95 and synaptophysin mRNA. Compared to acute irradiation, chronic exposure of ApoE null mice yields fewer consequences except for the increased microglia-mediated neuroinflammation. Exploring the function of ApoE in the hippocampus could have implications for developing therapeutic approaches to alleviate radiation-induced brain injury [38].

Further studies are required to determine whether changes in miR-124b-3p and miR-489-3p also occur in the blood and hippocampus following chronic irradiation at the same dose rate (1.2 mGy/h) but with varying cumulative doses, thereby clarifying whether these responses are specific to dose rate or cumulative dose.

### 4.4. Limitations

This study has several limitations that should be acknowledged. First, the sample size, while adequate for detecting the reported effects, is relatively modest. We acknowledge that the inclusion of all available male pups from multiple litters, without a split-litter design, may introduce potential litter effects that could not be statistically modelled due to sample size constraints. Larger cohorts would allow more detailed stratification of responses and increase statistical power for detecting subtle effects. However, blinding of all outcome assessments minimizes the risk of observer bias, and the use of multiple dams per group helps mitigate the influence of any single dam on the overall results. Second, although we identified two miRNA/mRNA axes with plausible functional roles, the causal relationships between these molecular changes and the observed behavioural phenotype have not been experimentally validated. Future studies using in vivo manipulation of these pathways (e.g., miRNA mimics or inhibitors, or conditional knockout of target genes) would be necessary to establish causality. Third, the behavioural assessments were conducted at a single time period, i.e., from P497 to P529. Longitudinal behavioural testing would provide insights into the temporal evolution of radiation-induced behavioural changes. Fourth, while we identified blood-based biomarkers, their specificity and sensitivity for detecting radiation exposure in other contexts or species remain to be determined. Fifth, the absence of analogous chronic low-dose-rate radiation exposure studies in the literature precludes a comprehensive comparison and discussion of our findings.

### 4.5. Summary

This study provides crucial experimental evidence (Table 1) for an important public health debate. It demonstrates that the health risks from chronic low-dose-rate exposure (like that following a nuclear accident) are not simply a scaled-down version of the risks from acute, high-dose exposure. The finding that a high cumulative dose caused limited pathology suggests that more research is needed to define dose-rate-dependent biological thresholds. This would provide a scientific basis for more balanced and effective public health decisions after nuclear incidents.

## Figures and Tables

**Table 1:** Summary of key findings.

Observation	Key Finding	Context and Significance
Behaviour	Anxiety-like behaviour (males only).	Differs from acute high-dose effects; suggests subtle, sex-specific outcomes.
Cellular Pathology	Persistent DNA damage in brain granule cells. No change in neurogenesis, cell counts, or inflammation.	Shows damage without major pathology, differing from acute high-dose-exposure effects.
Molecular Pathways	Dysregulation of miR-466i-5p/Tfcp2l1 (maladaptive) and miR-101a-5p/BMP6 (protective).	Proposed mechanisms linking DNA damage to anxiety, offering potential therapeutic targets.
Biomarkers	Two miRNAs (miR-124b-3p, novel-miR489-3p) were altered in the brain and blood of both sexes.	Identifies promising candidate biomarkers for detecting chronic radiation exposure.

## Data Availability

The data presented in this study are available on request from the corresponding authors.

## Supplementary Materials

The following supporting information can be downloaded at: https://www.mdpi.com/article/10.3390/cells15080711/s1, Supplementary S1–S11: all primer sequences used for RT-PCR validation; the sequences of inserted UTR fragments; the mutated seed-region changes for the luciferase assay; and the differentially expressed mRNAs and miRNAs in the hippocampus and blood of male and female mice used for Venn analysis in Figure 8A–G.

## References

[1] Tang F.R. (2024). Health effect of low-dose-rate irradiation with cumulative threshold dose: A promising area to explore in nuclear emergency and environmental contamination. Cells.

[2] Sutou S. (2025). Questioning the Linear No-Threshold Model (LNT): Lessons From Hiroshima/Nagasaki and Fukushima. Dose-Response.

[3] Sutou S. (2018). Low-dose radiation from A-bombs elongated lifespan and reduced cancer mortality relative to unirradiated individuals. Genes. Environ..

[4] David E., Wolfson M., Fraifeld V.E. (2021). Background radiation impacts human longevity and cancer mortality: Reconsidering the linear no-threshold paradigm. Biogerontology.

[5] Baker R.J., Chesser R.K. (2000). The Chernobyl nuclear disaster and the subsequent creation of a wildlife preserve. Environ. Toxicol. Chem..

[6] Mihok S.J. (2004). Chronic exposure to gamma radiation of wild populations of meadow voles (*Microtus pennsylvanicus*). Environ. Radioact..

[7] Gulay K.C.M., Tanaka I.B., Komura J., Tanaka S. (2018). Effects of continuous gamma-ray exposure in utero in B6c3f1 mice on gestation day 18 and at 10 weeks of age. Radiat. Res..

[8] Tanaka I.B., Nakahira R., Komura J.I., Tanaka S. (2022). Cause of Death and Neoplasia in B6C3F1 Mice Exposed in Utero to Low- and Medium-Dose-Rate Gamma Rays. Radiat. Res..

[9] Tang F.R., Tanaka I.B., Wang H., Lau S., Tanaka S., Tan A., Takai D., Abe A. (2024). Effects of continuous prenatal low dose rate irradiation on neurobehavior, hippocampal cellularity, messenger RNA and microRNA expression on B6C3F1 mice. Cells.

[10] Wang H., Lau S., Tan A., Tang F.R. (2024). Chronic low-dose-rate radiation-induced persistent DNA damage and miRNA/mRNA expression changes in the mouse hippocampus and blood. Cells.

[11] Wang H., Ma Z., Shen H., Wu Z., Liu L., Ren B., Wong P., Sethi G., Tang F.R. (2021). Early Life Irradiation-Induced Hypoplasia and Impairment of Neurogenesis in the Dentate Gyrus and Adult Depression Are Mediated by MicroRNA- 34a-5p/T-Cell Intracytoplasmic Antigen-1 Pathway. Cells.

[12] Li B., Dewey C.N. (2011). RSEM: Accurate transcript quantification from RNA-Seq data with or without a reference genome. BMC Bioinform..

[13] Love M.I., Huber W., Anders S. (2014). Moderated estimation of fold change and dispersion for RNA-seq data with DESeq2. Genome Biol..

[14] Ma Z., Liu T., Huang W., Liu H., Zhang H.M., Li Q., Chen Z., Guo A.Y. (2016). MicroRNA regulatory pathway analysis identifies miR-142-5p as a negative regulator of TGF-beta pathway via targeting SMAD3. Oncotarget.

[15] Baker R.J., Hamilton M.J., Van Den Bussche R.A., Wiggins L.E., Sugg D.W., Smith M.H., Lomakin M.D., Gaschak S.P., Bundova E.G., Rudenskaya G.A. (1996). Small mammals from the most-radioactive sites near the Chernobyl nuclear power plant. J. Mammal..

[16] Rodgers B.E., Baker R.J. (2000). Frequencies of micronuclei in bankvoles from zones of high radiation at Chornobyl, Ukraine. Environ. Toxicol. Chem..

[17] Wickliffe J.K., Chesser R.K., Rodgers B.E., Baker R.J. (2002). Assessing the genotoxicity of chronic environmental irradiation by using mitochondrial DNA heteroplasmy in the bank vole (*Clethrionomys glareolus*) at Chornobyl, Ukraine. Environ. Toxicol. Chem..

[18] Ross H.A. (1986). Genetic changes in an irradiated population of wild meadow voles (*Microtus pennsylvanicus*). Can. J. Zool..

[19] Kühne M., Rothkamm K., Löbrich M. (2002). Physical and biological parameters affecting DNA double strand break misrejoining in mammalian cells. Radiat. Prot. Dosim..

[20] Liao B., Dong S., Xu Z., Gao F., Zhang S., Liang R. (2020). LncRNA Kcnq1ot1 renders cardiomyocytes apoptosis in acute myocardial infarction model by up-regulating Tead1. Life Sci..

[21] Wang X., Li Z., Du Y., Xing Y., Guo Y., Zhang Y., Guo R., Gong W., Nie S., Wang X. (2022). lncRNA Mirt1: A critical regulatory factor in chronic intermittent hypoxia exaggerated post-MI cardiac remodelling. Front. Genet..

[22] Zhai X., Liu R., Li J., Wang F., Liu L., Wei S., Bian Y., Pang J., Xue M., Qin D. (2022). LincRNA-p21 upregulates nuclear orphan receptor Nr4a2 and aggravates myocardial ischemia/reperfusion injury via targeting miR-466i-5p. Int. Heart J..

[23] Carvelli A., Setti A., Desideri F., Galfre S.G., Biscarini S., Santini T., Colantoni A., Peruzzi G., Marzi M.J., Capauto D. (2022). A multifunctional locus controls motor neuron differentiation through short and long noncoding RNAs. EMBO J..

[24] Zhu J., Chen Y., Ji J., Wang L., Xie G., Tang Z., Qu X., Liu Z., Ren G. (2022). Microglial exosomal miR-466i-5p induces brain injury via promoting hippocampal neuron apoptosis in heatstroke. Front. Immunol..

[25] Chen J.X., Zhi J.W., Wang Y.P., Ning B. (2023). LncRNA-PEAK1 promotes neuronal apoptosis after intracerebral hemorrhage by miR-466i-5p/caspase 8 axis. Heliyon.

[26] Matsubara K., Hirota M., Kajiwara K., Senga H., Matsui S., Marutani M., Seki Y. (2025). Lineage-specific enhancer insertions regulatePrdm14 to drive the rapid transition from naïve to formative pluripotency in rodents. Development.

[27] Tomizawa K., Matsui H., Kondo E., Miyamoto K., Tokuda M., Itano T., Nagahata S., Akagi T., Hatase O. (1995). Developmental alteration and neuron-specific expression of bone morphogenetic protein-6 (BMP-6) mRNA in rodent brain. Mol. Brain Res..

[28] Martinez G., Carnazza M.L., Di Giacomo C., Sorrenti V., Vanella A. (2001). Expression of bone morphogenetic protein-6 and transforming growth factor-beta1 in the rat brain after a mild and reversible ischemic damage. Brain Res..

[29] Nonner D., Barrett E.F., Kaplan P., Barrett J.N. (2001). Bone morphogenetic proteins (BMP6 and BMP7) enhance the protective effect of neurotrophins on cultured septal cholinergic neurons during hypoglycemia. J. Neurochem..

[30] Crews L., Adame A., Patrick C., DeLaney A., Pham E., Rockenstein E., Hansen L., Masliah E. (2010). Increased BMP6 levels in the brains of Alzheimer’s disease patients and APP transgenic mice are accompanied by impaired neurogenesis. J. Neurosci..

[31] Sun L., Guo C., Wang T., Li X., Li G., Luo Y., Xiao S. (2014). LIMK1 is involved in the protective effects of bone morphogenetic protein 6 against amyloid-β-induced neurotoxicity in rat hippocampal neurons. J. Alzheimer’s Dis..

[32] Zhang Z., Trautmann K., Artelt M., Burnet M., Schluesener H.J. (2006). Bone morphogenetic protein-6 is expressed early by activated astrocytes in lesions of rat traumatic brain injury. Neuroscience.

[33] Yin J., Hu N., Yi L., Zhao W., Cheng X., Li G., Yang N., Li G., Ding D. (2021). Identification of Ferroptosis biomarker in AHH-1 lymphocytes associated with low dose radiation. Health Phys..

[34] Hall J., Jeggo P.A., West C., Gomolka M., Quintens R., Badie C., Laurent O., Aerts A., Anastasov N., Azimzadeh O. (2017). Ionizing radiation biomarkers in epidemiological studies—An update. Mutat. Res. Rev. Mutat. Res..

[35] Duale N., Eide D.M., Amberger M.L., Graupner A., Brede D.A., Olsen A.K. (2020). Using prediction models to identify miRNA-based markers of low dose rate chronic stress. Sci. Total Environ..

[36] Iinuma K., Kawakami K., Mizutani K., Fujita Y., Yamaguchi T., Ito M., Kumano T. (2021). miRNA-93 in serum extracellular vesicles before and after low dose rate prostate brachytherapy. Anticancer Res..

[37] Chen Y., Gong Y., Qin H., Wei S., Wei Y., Yu Y., Lin X., Shuai P., Wang T., Guo C. (2024). MDM2-p53 mediate a miR-181c-3p/LIF axis to regulate low dose-rate radiation-induced DNA damage in human B lymphocytes. Ecotoxicol. Environ. Saf..

[38] Casciati A., Pasquali E., De Stefano I., Braga-Tanaka I., Tanaka S., Mancuso M., Antonelli F., Pazzaglia S. (2024). Role of apolipoprotein in the hippocampus and its impact following ionizing radiation exposure. Cells.

