# Supplementary material for: Cellular, Molecular, and Behavioural Sequelae of Early-Life Continuous Low-Dose-Rate Irradiation in Mice
Source: Cells. 2026 Apr 17;15(8):711. doi: 10.3390/cells15080711 (PMC13114697; doi:10.3390/cells15080711)
Supplement: Supplementary file 1 [file cells-15-00711-s001.zip › Suppl 11-Table 4.pdf]

Table 4. Summary of Luciferase Reporter Assay

Gene	Construct	Mimic	Normalized Ratio (Mean ± SD)	Repression	p-value	Cohen's d	Effect Size
Bmp6	WT	miR-101a	0.29 ± 0.03	71%	<0.001	73.3	Extremely large
Bmp6	Mut	miR-101a	0.99 ± 0.05	—	0.78	0.26	Small
Igf2	WT	miR-466p	1.03 ± 0.02	(+3%)	0.048	-1.00	Large (reverse)
Igf2	Mut	miR-466p	1.06 ± 0.04	(+6%)	0.008	-1.52	Very large (reverse)
Six3	WT	miR-466i	0.96 ± 0.02	4%	0.042	1.04	Large
Six3	Mut	miR-466i	1.00 ± 0.02	—	0.83	0.21	Small
Tfcp2l1	WT	miR-466i	0.08 ± 0.01	92%	<0.001	14.0	Extremely large
Tfcp2l1	Mut	miR-466i	1.01 ± 0.04	—	0.67	-0.16	Negligible
Vector	psiCHECK-2	mimics mix	1.04 ± 0.04	—	0.22	—	—

p-values were calculated using two-tailed unpaired t-tests comparing each miRNA mimic group to its respective mimics NC control. Cohen's d values indicate effect size magnitude: 0.2 = small, 0.5 = medium, 0.8 = large, >1.2 = very large, >2.0 = extremely large.
