# Supplementary material for: Cellular, Molecular, and Behavioural Sequelae of Early-Life Continuous Low-Dose-Rate Irradiation in Mice
Source: Cells. 2026 Apr 17;15(8):711. doi: 10.3390/cells15080711 (PMC13114697; doi:10.3390/cells15080711)
Supplement: Supplementary file 1 [file cells-15-00711-s001.zip › Suppl-10-3UTR and seed sequence.pdf]

1. **Bmp6 3'UTR** (NM_007556.4)
GTTGAAGCTGGTGTGTGTGTgtgggtgggggcatggttctgccttggattcctaacaacaacatctgccttaaaccacgaacaaca
gcacagcgaagcgggatggtgacacacagagggatcgtgacacgcagacacatctcccgctggtgccttacccacggaggcttttatgaggacct
tgtcaagggctttcccagttcctaactgagcagttgctggtctgcaggaagctggaaggcttgtagtacaggcctggaaactgcagttacctaatgttc
gcctcccccaaccccgcccggagtagttttagcttttagatctagctgcttgtggtgtaagtagagagtaaacttgaaggaatattaaatatccctgggtt
gaaagacccggtggtggctctacagcacccatcccagggagatttttgcagacatccgaatggaggggagaagggcactctttcaggttccattcc
cagcaagggcagctcacacaggacctgcagcctggccatcagcaggctctgtggaggtgccttctgtctactgttgtagttacgtgttttgtgttgact
ctcggtggtgtgagaatgtactaatctctgtcaagacaaactgtagcatttccaccccatcctcctccctccctcacagaattctttactagaacccaaag
tagacagaccgtactttgtggcagagccatgaagcatctgccgtagccgtcatttaagccacagaacagcagaatttgattctacatctcgtgggcag
cctggctggacatgcgtgttaacatcaggaacactggctccaacatcggctctgtaacccctaactctgctctttgccttttccctacctaagcattccat
ggctcagggctagagtctaaagccgaaacccagagagggaagggccctgcctgtaagaatgggtttGTCAAGGTGAGCATGCTG
TG
seed seq 204-209 taactg mutated to GGCACT

2. **Igf2 3UTR** （NM_001122736.2）
TGCAATGTAGTACCATCaatctgtgacctcctcttgagcagggacagttccatcacgtcccacactaagatctctctgctccactccctc
cccaggtttctccttacccacccccatgccccgcctccccacatcaggctgttccccttgccccacaccatcgggcaaggggatctcagcagttctaa
aaaaccaaatttgattggctctaaacaacccaattgacaccccccaaattatatattcacattaaaaaaaactttaaaaactacagtccctttaaaaacaa
attggcttttaagaaattccataactgataatctaaaaattaaataactaaagaaattaattggctaaaaacatactaaaaattaattggcttaaaaacaatt
ggcaaaatcaaataattcggcgcccccccccttcatcttctttccatttggatctttagtcaaattggctaagacttggatcccagaacccaagaagaa
aggaaggggacccaaaattttgcaggtggcatgtcattgcttcagtgccctctccttatcccaagcacctttagtgtaaagctggctgagaacaacaat
agccgcccaaactctttcttcactggtcattccatcacaaatgtcccccatgtcaccaaggggggctgggtgaaagaacccaaggagaagaacagaa
catgaaaactggaaatagaacttaattggcacaagcccccagtcccaaaatctcacttttccctacctaccctaaaaagcacatgattatactcacacc
cacaggcattaacacacacatacacataacacatatgcacacacacacacacacacacacacacacacacagaggtatgcacacccacataatt
ggatgaaaacattgaaatggctgaacaacttcgatttgaaccacattccccaaaacaaggcccatcccaaatttcctgagcagtttgcatggtttgagc
tctcctcccaatccatctagtttctgctgctgcttattggctctatgtgtgaccaggctgctagttctgtctttctcagtgagggaggtgttctagcaaggct
caattccatctaagcacctgtccacatggtgagcccggtgctccccaaccacccccgccatgaggaccaatgactagccagtgtagaataagtttgg
ccagataaggagatggcactgccaagtgatacatgctgcccaagtaACCTGACCCCTAGTTGTGCT
seed seq 800-807 acacacat mutated to GTCAGTGC

3. **Six3 3UTR (**NM_011381.4)

ATCCCTAGAACAAACCGAAAtcaggatacacagacagatacacacacaagtccatacacactcccaccccagccaaaaatatatt
taaaaaaaaacaagaaaaataaattaacctcaaaccatcaacaaccccaccaccacctactaccacggccaccccaaaggaccgcgacgccaac
agacagtcacaaacgctgatgttgcgggcagaaagcataaaagaggtgacaattgtatactttctaggacaagcacggcttctccttttggttcccac
ggacccggcaccccacctgcatgacgatttatttgtatctgggaaaatattctctctagtttaaaatgatttaaagagactatacaaaaaaacaaaaaca
aaaacaaaaaaaaaacaccaccaccgccgctgcgacaagacgacaaaaagcagacaaaaagaaaaaaatctgacctctccgttttgaatttgttgtt
taaaaaaaaaaaaggaaagaaagataggaaaaaactcctcgagagaaatagcaaatgtttctaccttctgttgctcttttctcctttggccctctctctct
ctctctctctctctctctctctctctctctctctctctctctgtttttaagtccaagtattggtcaaacaaaatgcaatcctctgtttttgttcagccgacaat
cattttcttcgtaagcacctttttctctccactctgtcactgcctGTGTGGGTACTGGTTATAAATG

seed seq 129-136 acacacac mutated to GAGTGTCT

4. **Tfcp2l1 3UTR** (NM_023755.2)
GCAGCAGCGCCTGCACCTGTcccctgccccatggatccctggctgtgaaaggtgcaccactgtcagctgctgtctcacctgacaa
gctcagactaggagggactctacccagaccaggaaagctacagacaaccatggacacaaagtctggtgtacagaagaaagccagcggttcccttc
tcctttcctcttggcttttaggtttttaagtttcttgttctgtttcctgggcagatagtggggatgtgcatctcttaaactctgagccctgcagttggagggtcc
tttgatgtattggtccttcctgtctcctctcttatgtcaccctgattcctgcttctttttcttgagtcactggcttcatggctagctctgccaatctcacaagggc
tccacacacagttctcctcctaatactgacttcctttctcaggaccctggtagccatgtctctcctgttcttggatgacttgataagactggagctatttattt
gtgccttccatttctttctcagtcgccttgtctctaaggatctgctacattcaagcccagagtctctgcattgacatggtagatggcacagtgtacctatgt
gacctctcattgtgtattagttccttttctgttgctgtgataagatactctgacaaaggcaacttgataaagaaagggcttgttttggcttagagttttgtagg
gattatggtggatattatggtgaggaaggtgccaaacagaaagctagctgatctcattttcatacacgtaggaagcagcacagagagaaagagcaa
gagcaggaaatggtacaagccttcaaggaccaccaaccccagtgacttacttcctccagcaagactgcatcctctaaaagccccataacctcagca
gactacatcaccaactgggaccacgtgttcaaatacataagcccatgggatacatttctcattcaagccacacattttgcccatgtgacctcccacatttt

ctagttagagatgctatggactaggagatgacatgtgacttcctcaccagggaagagaggtgaggacagaatgattcctcatggaccactactgtgta
ttctcatttctgttgagcaaagagtactaagaggtgaagattctgttatccagtggccttgaccatccattctagacatttgggttggggtatgtctcagta
gatccctccctggcaagtatgaggccctgggcaccatcccagcgctacagcaggaggtacttactgaattctctttttcagACAACCTCAGC
TAACCACTG

seed seq 394-400 cacacac mutated to AGAGATT
