# Supplementary material for: Cellular, Molecular, and Behavioural Sequelae of Early-Life Continuous Low-Dose-Rate Irradiation in Mice
Source: Cells. 2026 Apr 17;15(8):711. doi: 10.3390/cells15080711 (PMC13114697; doi:10.3390/cells15080711)
Supplement: Supplementary file 1 [file cells-15-00711-s001.zip › Suppl 1-Table 1.pdf]

Table 1. Primer sequences for mRNA qRT-PCR.

mRNA	Sequences
Bmp6 forward	TGACACACAGAGGGATCGTG
Bmp6 reverse	CACCACCGAGAGTCAACACA
Ntn4 forward	CTACCGCGACCTCAGAAGAC
Ntn4 reverse	GAGTGTCGGAGGGCAGAAAA
H3c15 forward	CCCGCTCCATAGCTCTAGGT
H3c15 reverse	GGTGGTGAACCTACTTCGGG
Sgms2 forward	TCCTTCGCCGCATCTTGTAG
Sgms2 reverse	CCCTTTCGTAACCCGTTGGA
Rab11fip1forward	GGGCTTCAGGAGGAAGCATT
Rab11fip1 reverse	GAGGCCTCGGCTTCACAG
Zic4 forward	GCAGCTGGTCTTAGGGGTTT
Zic4 reverse	GGTGTCCACAGCTGCTACTT
Gpx8 forward	GAGACTCCCAGGATCGTCCAA
Gpx8 reverse	GATTGCACGGAAAAGCCAGG
Tent5a forward	CTCCAGGACTGACCAAGGC
Tent5a reverse	CGGACACCTATGCCCTTCTC
Igf2 forward	CCGTACTTCCGGACGACTTC
Igf2 reverse	GAGGGAGTGGAGCAGAGAGA
Six3 forward	AGGATGCCAAATGTGAGTGGA
Six3 reverse	GGAGAGCAACCGCATTTCAG
Tfcp2l1 forward	CTGCCCATCTTCAAGCAGGA
Tfcp2l1 reverse	CATGCTTCCTGGGGGTGAAT
Tnks forward	AACTGAACCGTGAACCTCCA
Tnks reverse	ATAGGTGGGTCACCACGAGA
Naaladl2 forward	GCCAACACTCAGGGCAAAAG
Naaladl2 reverse	TGAGTTACCTGGCCTTGCAG
Sh3bgrl2 forward	TGTCTGCCTGACTTCTCTTCCTT
Sh3bgrl2 reverse	GGTCTCCGTAGCAAGACTGT
Hivep3 forward	CCCACCATCCCCACTGAAAG
Hivep3 reverse	GGCAACCCGGGCTCCTTTAT
Nts forward	GCAAGTCCTCCGTCTTGGAA
Nts reverse	CCGGGCTGTTCACGTTATTT
Ddi2 forward	GACCTTTTCCCTCCAGGTCG
Ddi2 reverse	GGTGAGAGGCCTTTCTGCAT
Mcm10 forward	TAACTAGGGCAGAGGGGCTC
Mcm10 reverse	AGAGTGGCCAAACGTTCCTC
Aif1 forward	ATCTGGGGAAAGCCACTGTC
Aif1 reverse	GACGCTGGTTGTCTTAGGCT
Trpv6 forward	GAGACACAAGCCCAGCAGAT
Trpv6 reverse	CCATTGCAGCCTCCAGGTTA
Vcam1 forward	ACTTTCTAATTCATGGTAGAATGGC
Vcam1 reverse	CAATGAAGAAACAGGTCCCCG
Slc15a2 forward	CAGGGAACGAGCTTGGGAAT
Slc15a2 reverse	GCAGTTGTCTGGGGAAAGGA
Mlxipl forward	CCTGAGCATCTGCAGCCTC
Mlxipl reverse	ATGACAGCCTCAGGTTTCCG
Rdh5 forward	CTCAGCAGGGCATCTCATCC
Rdh5 reverse	CACTTGGCAACTTGCTGGAC
Mcpt4 forward	AGAATCTCTCTCCAAGCTGT
Mcpt4 reverse	GTAAGGGCGAGAATGTGGTC
Tnni3 forward	TGTCCTCGCCCCTTATCTCA

Tnni3 reverse	GGTTCCCCAGCCGCATC
Tppp3 forward	TAGAAGCCGGGTGGCATGG
Tppp3 reverse	GTTCTTTGTGGGAGCCCGTA
Rbpms forward	ATTGCCTCAAGAGGAGCAGG
Rbpms reverse	GGGCGGTCTATCTGACATGG
Cdkn2c forward	CCGGCACAGTACCTTCAGAG
Cdkn2c reverse	AGCTCAGGCTCTTCACTGCAA
S1pr5 forward	ACACCAAATGCCCAGCTTAC
S1pr5 reverse	AAGTCTCCTGTAACCGGCAC
