# Supplementary material for: Cellular, Molecular, and Behavioural Sequelae of Early-Life Continuous Low-Dose-Rate Irradiation in Mice
Source: Cells. 2026 Apr 17;15(8):711. doi: 10.3390/cells15080711 (PMC13114697; doi:10.3390/cells15080711)
Supplement: Supplementary file 1 [file cells-15-00711-s001.zip › Suppl 2-Table 2.pdf]

Table 2. Primer sequences for miRNA sequences for qRT-PCR.

Targets	Sequences
miR-101a-5p	TCAGTTATCACAGTGCTGATGC
miR-193a-3p	AACTGGCCTACAAAGTCCCAGT
miR-466c-3p	ATACATACACGCACACATAAGA
miR-466i-5p	TGTGTGTGTGTGTGTGTGTG
miR-466p-5p	CGTATGTGTGTGTACATGTACAT
miR-669f-3p	GCATATACATACACACACACGTAT
miR-1224-3p	CCCCACCTCTTCTCTCCTCAG
miR-1298-5p	TTCATTCGGCTGTCCAGATGTA
miR-3082-3p	CACATGGCACTCAACTCTGCAG
miR-6900-5p	CAG GAG AAG CCT AGA GCC GTC
miR-33-5p	GTGCATTGTAGTTGCATTGCA
miR-34c-3p	AATCACTAACCACACAGCCAGG
miR-301b-3p	CAGTGCAATGGTATTGTCAAAGC
miR-335-3p	TTTTTCATTATTGCTCCTGACC
miR-344f-5p	AGTCAGTCTCCTGGCTGGAGTC
miR-350-5p	AAAGTGCATGCGCTTTGGG
miR-450a-1-3p	GCATTGGGAACATTTTGCATAAAT
miR-551b-3p	GCGACCCATACTTGGTTTCAG
miR-666-3p	GGCTGCAGCGTGATCGCCTGCT
miR-7015-3p	TCTCACTGTCCTCTGCACTAG
miR-124b-3p	TCAAGGTCCGCTGTGAACACGG
miR-181b-5p	AACATTCATTGCTGTCGGTGGGTT
miR-206-3p	TGGAATGTAAGGAAGTGTGTGG
miR-212-5p	ACCTTGGCTCTAGACTGCTTACT
miR-296-5p	AGGGCCCCCCCTCAATCCTGT
miR-466b-3p	ATACATACACGCACACATAAGA
miR-350-5p	AAAGTGCATGCGCTTTGGG
miR-3074-5p	GTTCCTGCTGAACTGAGCCAGT
miR-6967-3p	TCATCTTTATCTCTCCCCAG
miR-7037-5p	AAGGTGGCCACAGGAGATCATGGT
