# Supplementary material for: Cellular, Molecular, and Behavioural Sequelae of Early-Life Continuous Low-Dose-Rate Irradiation in Mice
Source: Cells. 2026 Apr 17;15(8):711. doi: 10.3390/cells15080711 (PMC13114697; doi:10.3390/cells15080711)
Supplement: Supplementary file 1 [file cells-15-00711-s001.zip › Suppl 3-Table 3.pdf]

Table 3. Primer sequence for Luciferase assay.

Primer ID	Sequence (5'-3')	Notes
L1628	gactcatttagatcctcacac	psiCHECK2 Reverse (rev) sequencing primer
L1123	cctccacttcagccaggagg	psiCHECK2 Forward (fwd) sequencing primer
L3247	aattctaggcgatcgctcgagGTTGAAGCTGGTGTGTGTGT	fwd primers for mouse *Bmp6* 3'UTR with XhoI to psiCHECK2
L3255	attttattgcggccagcggccgcCACAGCATGCTCACCTTGAC	rev primers for mouse *Bmp6* 3'UTR with NotI to psiCHECK2
L3256	aattctaggcgatcgctcgagTGCAATGTAGTACCATC	fwd primers for mouse *Igf2* 3'UTR with XhoI to psiCHECK2
L3258	attttattgcggccagcggccgcAGCACAACTAGGGGTCAGGT	rev primers for mouse *Igf2* 3'UTR with NotI to psiCHECK2
L3259	aattctaggcgatcgctcgagATCCCTAGAACAAACCGAAA	fwd primers for mouse *Six3* 3'UTR with XhoI to psiCHECK2
L3260	attttattgcggccagcggccgcCATTTATAACCAGTACCCACAC	rev primers for mouse *Six3* 3'UTR with NotI to psiCHECK2
L3253	aattctaggcgatcgctcgagGCAGCAGCGCCTGCACCTGT	fwd primers for mouse *Tfcp2l1* 3'UTR with XhoI to psiCHECK2
L3254	attttattgcggccagcggccgcCAGTGGTTAGCTGAGGTTGT	rev primers for mouse *Tfcp2l1* 3'UTR with NotI to psiCHECK2
L3261	gggctttcccagttccGGCACTagcagttgctggtctgcaggaagctggaaggc	*Bmp6* MUT Primers
L3262	gaccagcaactgctAGTGCCggaactgggaaagcccttgacaaggtcctcataaa	
L3263	acccacaggcattaacGTCAGTGCacacataacacatatgcacacacacacacac	*Igf*2 MUT Primers
L3264	tatgtgttatgtgtGCACTGACgttaatgcctgtgggtgtgagtataatcatgtg	
L3265	tcaggatacacagacagatGAGTGTCTaagtccatac	*Six3* MUT Primers
L3266	tatatttttggctggggtgggagtgtgtatggacttAGACACTCatctgtctgt	
L3267	tctcacaagggctcAGAGATTagttctcctcctaatactgacttcctttctcagga	*Tfcp2l1* MUT Primers
L3268	ttaggaggagaactAATCTCTgagcccttgtgagattggcagagctagccatgaag	
